# Analysis of a slow-growing line reveals wide genetic variability of carcass and meat quality-related traits

**DOI:** 10.1186/1471-2156-13-90

**Published:** 2012-10-23

**Authors:** Marie Chabault, Elisabeth Baéza, Vérane Gigaud, Pascal Chartrin, Hervé Chapuis, Maryse Boulay, Cécile Arnould, François D’Abbadie, Cécile Berri, Elisabeth Le Bihan-Duval

**Affiliations:** 1 Institut National de la Recherche Agronomique (INRA), UR83 Recherches Avicoles, F-37380, Nouzilly, France; 2 Institut Technique de l’Aviculture (ITAVI), Centre INRA de Tours, F-37380, Nouzilly, France; 3 Syndicat des Sélectionneurs Avicoles et Aquacoles Français (SYSAAF), Centre INRA de Tours, F-37380, Nouzilly, France; 4 Institut National de la Recherche Agronomique (INRA), UMR85 Physiologie de la Reproduction et des Comportements, F-37380, Nouzilly, France; 5 CNRS, UMR7247, F-37380, Nouzilly, France; 6 Université François Rabelais de Tours, F-37041, Tours, France; 7 IFCE, F-37380, Nouzilly, France; 8 SASSO, F-40630, Sabres, France

**Keywords:** Chicken, Slow-growing line, Meat quality, Behaviour at slaughter, Genetic parameters

## Abstract

**Background:**

Slow-growing lines are widely used in France for the production of high quality free-range chickens. While such production is mainly dedicated to the whole carcass market, new prospects are opening up for the development of cuts and processed products. Whether the body composition and meat quality of slow-growing birds can be improved by selection has thus become an important issue. The genetic parameters of growth, body composition and breast meat quality traits were evaluated in relation to behaviour at slaughter in a large pedigree population including 1022 male and female slow-growing birds.

**Results:**

The heritability coefficients (h^2^) of body weight and body composition traits varied from 0.3 to 0.5. Abdominal fat percentage was genetically positively correlated with body weight but negatively correlated with breast muscle yield. The characteristics of the breast meat (*i.e.*, post-mortem fall in pH, colour, drip loss, shear-force and lipid content) were all heritable, with h^2^ estimates ranging from 0.18 to 0.48. The rate and extent of the fall in pH were under different genetic control. Strong negative genetic correlations were found between the ultimate pH and the lightness, yellowness and drip loss of the meat. Wing flapping on the shackle line was significantly heritable and exhibited marked genetic correlations with the pH at 15 min post-slaughter and the redness of the meat. The genetic relationships between meat quality traits, body weight and body composition appeared slightly different between males and females.

**Conclusion:**

This study suggested that there are a number of important criteria for selection on carcass and breast meat quality in slow-growing birds. Selection for reduced abdominal fatness and increased breast muscle yield should be effective as both traits were found to be highly heritable and favourably correlated. Substantial improvement in meat quality could be achieved by selection on ultimate pH which was highly heritable and strongly correlated with the colour and water-holding capacity of the meat. Moreover, this study revealed for the first time that the behaviour at slaughter is partly genetically determined in the chicken.

## Background

The production of Label Rouge chicken began in France in the sixties, with the breeding of slow-growing lines well adapted to free range conditions and slaughtered at a more mature age (a minimum of 81 days) than conventional fast-growing lines (usually slaughtered between 35 and 42 days) [1,2]. It has been shown that the tenderness and juiciness of the meat decreases while flavour intensity increases when birds get older [3-7]. The higher sensorial quality of the meat and the strict regulations applied to the rearing conditions (low density, free access to outdoors, etc.) of Label Rouge chickens have contributed to the success of this product which is now extensively present in the whole carcass market in France (56% of the market in 2011). However, the market share of Label Rouge chicken for cuts and processed products is still limited (11% of cuts in 2011). In the long term this could become critical for the maintenance of such alternative products since there is increased consumption of cuts and processed products in most developed countries compared to whole carcasses. In order to estimate to what extent Label Rouge chickens could be adapted by selection to these market demands, we evaluated the genetic parameters of breast meat quality traits in relation to body weight and body composition in a slow-growing line. As a phenotypic relationship between the behaviour of these birds at slaughter and breast meat quality traits had previously been observed [8], the genetic relationships between these traits were also evaluated.

## Methods

### Animals

The birds originated from a slow-growing line selected by the SASSO breeding company (Sabres, France) since 1994. Birds were naked-neck chickens with yellow shanks. A large pedigree population was constituted of around 1022 male and female birds originating from 10 sires and 100 dams. Birds were reared in three successive batches (mixed sex) under similar free-range conditions. They had access to an outdoor area after 6 weeks of age. The bird density in the poultry house was 11 chickens per m^2^, and outside it was 2 chickens per m^2^ as required by the French legislation on Label Rouge production. Chickens were fed *ad libitum*, first with a starting diet from day 0 to 21 (Metabolisable Energy: 2740 kcal/kg, Proteins: 201.9 g/kg, Lysine: 10.9 g/kg, Methionine: 4.7 g/kg), then with a growing diet from day 22 to 56 (Metabolisable Energy: 2863 kcal/kg, Proteins: 169.5 g/kg, Lysine: 9.4 g/kg, Methionine: 3.9 g/kg), followed by a finishing diet from day 57 to 84 (Metabolisable Energy: 2930 kcal/kg, Proteins: 159.5 g/kg, Lysine: 7.9 g/kg, Methionine: 3.7 g/kg). At 3, 6 and 9 weeks of age, birds were individually weighed and then at 12 weeks of age, birds were weighed and slaughtered after 7 hours’ feed withdrawal. Birds did not undergo transport before they were slaughtered in the experimental slaughter plant (INRA, Pôle d’Expérimentation Avicole de Tours, F-37380 Nouzilly, France). Duration of wing flapping (WF) on the shackle line was individually measured as previously described by Debut et *al.*[9]. Before sacrificing by ventral neck cutting, birds were electrically stunned (120 Hz AC, 80 mA/bird, 5 s) in a water bath, bled for 3 min, and scalded at 51°C for 3 min. After removal of the gut, whole carcasses were air chilled (airflow of 7 m^3^/s) and stored at 2°C until the next day.

### Carcass and meat characteristics

At 15 min post-mortem, the pH (pH_15_) was measured in the right Pectoralis *major* muscle as described by Berri *et al.*[8]. Samples of Pectoralis *major* muscle (about 60 g) were frozen at −20°C for lipid determination. After thawing for 12 h at 4°C, samples were ground and intramuscular fat content (IMF) was measured by near-infrared spectroscopy on a Nirflex N-500 (Buchi, Rungis, France) as described by Chartrin *et al.*[10].

Carcasses were dissected 24 h post-mortem and the left breast meat (Pectoralis *major* plus *minor*), abdominal fat and leg weights were measured as described by Marche [11]. Yields were also calculated as percentages of body weight at slaughter. All measurements of meat quality were performed on the left Pectoralis *major* muscle. At 24 h post-mortem, the pH (pHu) was measured by direct insertion of the electrode in the muscle. Colour was measured on the upper ventral side of the muscle by using a Miniscan Spectrocolorimeter (Hunterlab, Reston, VA, USA). Colour was measured by the CIELAB trichromatic system according to lightness (L*), redness (a*) and yellowness (b*) values. The water holding capacity of meat was estimated by measuring drip loss of the raw meat after storage. The breast Pectoralis *major* muscle was weighed 24 h postmortem and immediately placed in a plastic bag, hung from a hook, and stored at 2°C for 5 d. After hanging, each sample was wiped with absorbent paper and weighed again. The difference in weight corresponded to the drip loss and was expressed as the percentage of the initial muscle weight. After drip-loss evaluation, each muscle sample was vacuum-packed, cooked in a water-bath at 85°C for 15 min, and cooled in crushed ice for 10 min. After cooling, the sample was unpacked then wiped with absorbent paper. The texture of cooked meat was evaluated with a Warner Bratzler shear test according to the recommendations of Pettracci and Baeza [12]. The meat was sheared using a single shear blade, with a triangular shaped hole, placed on an Instron Universal Testing Instrument (Instron 5543, Guyancourt, France). Blade thickness was 1.1 mm, the height and width at the base of the triangle were 52 and 61 mm, respectively, and the rate of travel of the blade was 80mm/min. Three adjacent strips of meat were cut from the medial portion of the P. major muscle so that the cross-section measured 10 x 10 mm and the muscle fibers ran parallel to the length of the axis for 30 mm. The samples were sheared at a right angle to the fibre axis. The parameter measured was the maximum force (N) recorded.

### Estimation of the genetic parameters

Descriptive statistics were calculated by the UNIVARIATE procedure of SAS software [13]. The dataset analysed included a total of 1022 observations for growth, body composition, breast meat quality traits and behaviour at slaughter. Heritability estimates and genetic correlations were computed in multitrait analyses by the Wombat software [14] using the REstricted Maximum Likelihood methodology and an animal model. This software accommodates high dimension issues with strongly correlated traits through reduced rank analyses. The model included the fixed effects of hatch and sex, the direct genetic effect of the animal and the environmental maternal effect in the case of body weight measured at 3, 6 and 9 weeks. Separate analyses by sex were also performed according to the same model but without sex effect. Approximate sampling errors of variance-covariance components were derived at convergence from the inverse of the average information matrix. Sampling errors of heritabilities or correlations were derived by first approximating the function by its first order Taylor series expansion, and then computing the variance of the latter. Five generations of ancestors were considered in the pedigree which included a total of 1489 animals. The distribution of wing flapping duration could not be considered as Gaussian. It was therefore classified in three categories, 1 when the bird did not flap (N=446), 2 when WF duration was less than 9 s (N=248) and 3 when WF duration was between 9 and 36 s (N=248). The heritability of WF and the genetic correlations with continuous traits were estimated after a series of two-trait analyses using a threshold model with an animal effect using TM software based upon Gibbs Sampling and developed by A. Legarra (INRA, Station d’Amélioration Génétique des Animaux, F-31326 Castanet-Tolosan Cédex).

## Results

### Phenotypic variability

Except for WF, the distribution of the traits analyzed was close to normal. As shown in Table 1, the coefficient of variation (CV) for body weight in the whole population remained fairly stable with age whereas it decreased when males and females were considered separately. CV for body weight at 12 weeks was about two-fold lower within each sex than in the whole population. The CV for breast meat yield was more moderate, while it was quite high for the percentage of abdominal fat especially in males. In contrast, the variation for percentage of leg was limited for both sexes (4.1 to 5.1). The CV for early and ultimate pH values was low (from 1.9 to 3%). The coefficients of variation for the other meat quality traits varied considerably and were relatively small for lightness, moderate for yellowness, shear force and intramuscular fat content but quite high for drip loss and redness of the meat.

**Table 1:** Body weight, body composition and meat quality-related traits

	**Males + Females**					**Males**					**Females**				
	**N**	**Mean**	**CV (%)**	**Min.**	**Max.**	**N**	**Mean**	**CV (%)**	**Min.**	**Max.**	**N**	**Mean**	**CV (%)**	**Min.**	**Max.**
**body weight and body composition**															
BW3 (g)	1022	415	14.4	234	603	515	432	14.4	234	603	507	397	12.8	247	545
BW6 (g)	1006	1127	12.8	710	1523	508	1208	11.1	749	1523	498	1045	9.7	710	1373
BW9 (g)	1000	1825	13.4	1193	2478	503	2006	9.4	1341	2478	497	1642	8.1	1193	2089
BW12 (g)	958	2678	16.3	1750	3852	473	3052	8.8	2188	3852	485	2314	8.5	1750	2842
BMY (%)	910	16.5	8.0	12.1	21.7	450	16.2	7.3	12.1	19.9	460	16.8	7.9	12.9	21.7
AFP (%)	914	4.0	39.1	0.6	8.6	451	3.2	42.9	0.6	8.1	463	4.9	26.1	0.6	8.6
LEGP (%)	912	25.7	5.0	21.4	30.8	452	26.4	4.1	23.4	30.1	460	25.0	5.1	21.4	30.8
**meat quality traits**															
pH15	921	6.61	3	5.98	7.03	458	6.62	2.7	5.98	6.97	463	6.6	3.3	5.98	7.03
pHu	931	5.80	1.9	5.42	6.17	459	5.81	1.9	5.45	6.17	472	5.78	1.8	5.42	6.1
L*	935	47.5	6.2	37.0	55.7	464	46.9	6.4	37.0	55.5	471	48.1	5.7	41.4	55.7
a*	940	−1.5	66	−4.9	2.3	466	−1.3	77.5	−4.9	2.3	474	−1.7	54.6	−4.1	1.6
b*	935	8.5	17	4.7	14.7	464	8.3	18.5	4.7	14.7	471	8.7	15.5	5	12.9
DL (%)	909	1.6	38.1	0.2	3.9	449	1.4	37.5	0.2	3.6	460	1.8	35.0	0.22	3.9
SF (N/cm^2^)	924	18.7	24.6	10.8	48.8	455	18.4	21.9	11.1	43.5	469	19.1	26.7	10.8	48.8
IMF (%)	693	1.0	22.1	0.6	2.1	340	1.0	23.9	0.6	2.1	353	1.1	20.5	0.6	2.0

BW3 = Body weight at 3 weeks; BW6 = Body weight at 6 weeks; BW9 = Body weight at 9 weeks; BW12 = Body weight at 12 weeks.

BMY = Breast meat yield; AFP = Abdominal fat percentage; LEGP = Leg percentage; pH15 = pH at 15 min post-mortem; pHu = ultimate pH.

L* = lightness; a* = redness; b* = yellowness; DL = drip loss; SF = shear force; IMF = Intramuscular fat content.

A significant effect of sex was found on all the traits except pH15: as expected, males were heavier than females as early as 3 weeks and throughout the rearing period (p<0.0001). The females were fattier at slaughter age (p<0.0001) and had a slightly higher breast meat yield (p<0.0001) and lower percentage of leg (p<0.0001) than the males. Significant differences were also observed in meat characteristics: the meat of females had a slightly lower pHu (p<0.0001) and redness (p<0.0001) than the males but a higher shear force (p<0.02), drip loss (p<0.0001), lightness (p<0.0001) and yellowness (p<0.0001). Lipid content of the breast meat was slightly higher for females than for males (p<0.005).

### Heritability estimates and genetic correlations in the whole population

#### Body weight and body composition traits

Heritability of body weight increased with age and ranged between 0.31 at 3 weeks to 0.46 at 12 weeks (Table 2). Genetic correlations between the measurements of body weight at the different ages were all positive and high when the ages were close (0.8-0.94) but more moderate between the early and the final body weight (only 0.50 between BW3 and BW12). Heritability for body composition traits (BMY, AFP, LEGP) was high (between 0.43 and 0.53). AFP was genetically positively correlated with body weight at the different ages but negatively correlated with BMY.

**Table 2:** Heritability estimates (on the diagonal) and genetic correlations (above the diagonal) for body weight, body composition and meat quality traits in the whole population

	**BW3**	**BW6**	**BW9**	**BW12**	**BMY**	**AFP**	**LEGP**	**pH15**	**pHu**	L*	a*	b*	**DL**	**SF**	**IMF**
BW3	0.31 ± 0.09	0.80 ± 0.08*	**0.7 ± 0.10**	**0.50 ± 0.15**	0.16 ± 0.20	**0.45 ± 0.17**	0.16 ± 0.19	0.10 ± 0.21	-0.03 ± 0.18	0.13 ± 0.19	**-0.41 ± 0.18**	**-**0.18 ± 0.21	**-**0.07 ± 0.21	0.15 ± 0.22	0.33 ± 0.24
BW6		0.31 ± 0.07	**0.94 ± 0.03**	**0.80 ± 0.08**	0.28 ± 0.19	**0.46 ± 0.16**	0.08 ± 0.19	-0.10 ± 0.21	-0.04 ± 0.18	0.04 ± 0.19	**-**0.27 ± 0.19	**-**0.32 ± 0.21	**-**0.01 ± 0.21	0.03 ± 0.23	0.29 ± 0.25
BW9			0.45 ± 0.08	**0.93 ± 0.03**	0.30 ± 0.17	**0.45 ± 0.14**	0.16 ± 0.18	-0.06 ± 0.19	-0.12 ± 0.17	0.15 ± 0.17	**-0.42 ± 0.16**	**-**0.28 ± 0.19	0.03 ± 0.20	0.08 ± 0.21	0.35 ± 0.23
BW12				0.46 ± 0.09	0.22 ± 0.17	**0.38 ± 0.15**	0.20 ± 0.17	-0.11 ± 0.18	-0.26 ± 0.15	0.24 ± 0.16	**-0.42 ± 0.16**	**-**0.20 ± 0.18	0.01 ± 0.19	0.18 ± 0.20	0.34 ± 0.22
BMY					0.46 ± 0.12	**-0.42 ± 0.14**	0.17 ± 0.17	-**0.41 ± 0.18**	0.13 ± 0.17	**-**0.19 ± 0.17	0.12 ± 0.18	0.06 ± 0.19	0.20 ± 0.19	**-**0.08 ± 0.21	**-**0.29 ± 0.23
AFP						0.53 ± 0.14	**-**0.24 ± 0.16	0.33 ± 0.17	0.15 ± 0.16	0.02 ± 0.17	**-0.46 ± 0.15**	**-0.43 ± 0.16**	**-**0.21 ± 0.19	**-**0.12 ± 0.20	0.32 ± 0.21
LEGP							0.43 ± 0.08	-0.08 ± 0.19	-0.18 ± 0.17	0.13 ± 0.17	**-**0.13 ± 0.18	**-**0.14 ± 0.19	0.04 ± 0.19	**0.62 ± 0.16**	0.25 ± 0.23
pH15								0.34 ± 0.08	-0.09 ± 0.18	0.16 ± 0.17	**-0.51 ± 0.14**	0.04 ± 0.20	**-**0.24 ± 0.19	**-0.44 ± 0.17**	**-**0.13 ± 0.24
pHu									0.48 ± 0.08	**-0.83 ± 0.07**	0.15 ± 0.17	**-0.53 ± 0.14**	**-0.68 ± 0.12**	**-**0.26 ± 0.18	**-**0.03 ± 0.21
L*										0.44 ± 0.10	**-**0.28 ± 0.16	**0.51 ± 0.14**	**0.64 ± 0.13**	0.34 ± 0.19	**0.42 ± 0.20**
a*											0.39 ± 0.10	0.05 ± 0.19	0.03 ± 0.19	0.22 ± 0.19	**-**0.20 ± 0.24
b*												0.30 ± 0.11	**0.47 ± 0.18**	0.02 ± 0.22	**-**0.33 ± 0.24
DL													0.30 ± 0.12	0.10 ± 0.22	**-**0.04 ± 0.25
SF														0.22 ± 0.08	**0.57 ± 0.21**
IMF															0.18 ± 0.07

BW3 = Body weight at 3 weeks; BW6 = Body weight at 6 weeks; BW9 = Body weight at 9 weeks; BW12 = Body weight at 12 weeks.

BMY = Breast meat yield; AFP = Abdominal fat percentage; LEGP = Leg percentage; pH15 = pH at 15 min post-mortem; pHu = ultimate pH.

L* = lightness; a* = redness; b* = yellowness; DL = drip loss; SF = shear force; IMF = Intramuscular fat content. * Significant genetic correlations are in bold.

#### Meat quality-related traits

Heritability of meat quality related traits varied between 0.18 (for IMF) to 0.48 (for pHu). pH15 and pHu were not genetically correlated (rg of −0.09). Significant genetic correlations were revealed between the fall in pH and meat characteristics: the greater the early rate of fall in pH the redder, the less tender the breast meat (rg of −0.51 between pH15 and a*, of −0.44 between pH15 and SF), but the lower the ultimate pH, the lighter, the more yellow and the more exudative the meat (rg of -0.83, -0.53 and -0.68 between pHu and L*, b* and DL, respectively). Significant positive genetic correlations were consistently found between L*, b* and DL of the meat (0.47-0.64). The results also revealed positive genetic correlations between the intramuscular fat content and lightness (rg of 0.42) as well as the shear force (rg of 0.57) of the meat.

#### Genetic correlations between growth, body composition and meat quality-related traits

The genetic correlations between body weight and body composition and the meat quality traits were generally low to moderate. However, meat redness exhibited a negative correlation with body weight (−0.42) and AFP (−0.46). AFP was also negatively related to yellowness (rg of −0.43) and BMY to pH15 (rg of −0.41). A marked positive genetic correlation was observed between LEGP and SF (rg of +0.62).

#### Behaviour at slaughter and meat quality

The estimated heritability of duration of wing flapping was 0.41 ± 0.09, with low to moderate genetic correlations (0.04-0.20 in absolute value) with BW12, BMY, AFP, LEGP, pHu, L*, b*, DL, SF, and IMF. By contrast, significant genetic correlations were found between WF and pH15 as well as a*, estimates being -0.71 ± 0.11 and 0.52 ±0.15, respectively.

### Heritability estimates and genetic correlations according to sex

The genetic parameters for male and female traits are reported in Additional file 1: Table S1 and Additional file 1: Table S2.

#### Body weight and body composition traits

Heritability of BW9, BW12, and BMY was higher in females (from 0.46 to 0.60) than in males (from 0.29 to 0.39) while it was the opposite for AFP. However, standard errors were greater than in the whole population, which was to be expected as the sample size was lower. The positive genetic correlation between body weight at the different ages and AFP was stronger in the males (between 0.43 and 0.60) than in the females (between 0.16 and 0.35, NS), while the negative genetic correlation between AFP and BMY was more marked in the females (−0.57) than in the males (−0.38, NS).

#### Meat quality in relation to body weight and body composition

Heritability of meat quality traits was similar in both sexes, except for L*, DL and IMF for which it was higher in the males (0.55, 0.43, and 0.25 respectively) than in the females (0.45, 0.31, and 0.16 respectively). Strong negative genetic correlations between body weight and AFP and meat redness (a*) were observed in males (around -0.75) whereas they were much lower and non-significant in females (between −0.28 and 0). As in the whole population, a negative genetic correlation between pH15 and BMY was observed in both the males (−0.52) and females (−0.43). The genetic correlations between L* and pHu and between L* and DL were lower (in absolute value) in the females (−0.78 and 0.36, respectively) than in the males (−0.90 and 0.84, respectively). Breast meat SF and LEGP remained positively correlated for both sexes (0.72 and 0.65 for males and females, respectively) whereas the correlation between SF and L* was significant in the females (0.57) but not in the males (0.10).

## Discussion

Because the minimal age of the animals required by the “label” organisation is 81 days, the “label” strains have to be chosen from a specific list which only contains slow-growing lines. Independently of the characteristics associated with reproduction, the selection of these lines includes several traits associated with the quality of the final product such as body weight (mainly to avoid genetic drift), body conformation (improvement of the breast angle and a better meat and bone yield), fattening (which has to be maintained rather low) as well as the thickness of the skin and the feed conversion [1,2]. This study was the first one dealing with the genetic aspects of carcass and meat quality related traits in a “label” strain. The small number of sire families considered in this study was a limitation regarding the accuracy of the genetic parameter estimates, especially the genetic correlations, which should be confirmed on a larger data set in order to find out the most relevant criteria of selection. However, one can underline the fact that several estimated correlations were consistent with previous ones obtained on conventional broiler lines, showing that we may be confident about the sign and the strength of these relationships.

Wide genetic variability for body weight and body composition traits was found in the slow-growing line used in the present study. It was associated with a fairly extensive phenotypic variability, especially for traits such as AFP. Whereas weight gain is not desirable in such a line, selection for reduced AFP and increased BMY could be valuable particularly since both traits are highly heritable and favourably (*i.e.* negatively) correlated, as already reported in several other chicken lines [15,16]. Improving body composition could have important economic benefits due to the expected gain in feed efficiency and cut yields. However, whether meat quality might be impaired is an important issue which may be addressed by estimating the genetic correlations between body composition and meat quality traits. According to our results, selection for decreasing carcass fatness by reducing AFP should not lead to any significant change in IMF, which is known to influence sensorial characteristics of the meat such as juiciness and flavour [7]. Similarly, the studies by Chen *et al.*[17] and Zerehdaran *et al.*[15] failed to demonstrate any significant correlation between the deposition of abdominal fat and that of intramuscular fat in breast muscle, whereas a positive genetic correlation was reported between abdominal and subcutaneous fat. On the other hand, our results indicated that selection for lower carcass fatness should increase the intensity of the red and yellow colour of breast meat, especially in males in which a strong genetic link between AFP and body weight was also found. Moreover, selection for greater muscle development of the bird, including the breast and leg, could have an impact on meat characteristics as revealed by the negative genetic correlation between BMY and pH15 and the positive correlation between LEGP and SF. Although some sensory tests would be needed, these genetic correlations do not support a marked deleterious effect of selection for higher breast meat yield and lower abdominal fatness on the sensorial quality of the meat. Moreover, no genetic correlation was found with the ultimate pH, a factor determining the technological quality of meat [8].

The genetic parameters of measurements of the technological quality of the meat (such as the pH, colour, drip loss or shear-force) were evaluated for the first time in a slow-growing line. As in previous studies on intermediate and fast-growing chickens [18,19], genetics explained a large part of the variability of these traits, with heritability estimates ranging from 0.22 to 0.48. Also in this slow-growing line, the ultimate pH of the meat was highly heritable and strongly genetically correlated with the colour and water-holding capacity of the meat. Acid meat defects, characterized by a low ultimate pH, pale colour and low water-holding capacity, have become a major problem in the United States and Europe where their frequency ranges between 5% and 47% within a flock [20]. A survey undertaken between 2007 and 2010 in various French slaughter plants showed that it affected not only standard but also alternative products. When compared with intermediate or fast-growing chickens used for standard production, the slow-growing birds used for Label Rouge production had the lowest ultimate pH [21], which was consistent with their higher glycogen reserve in breast muscle [9]. The incidence of meat exhibiting low pH (<5.7) was high in all types of production, but the highest proportion (around 50%) occurred in the free-range Label-Rouge production. The present study indicates that substantial improvement of the technological quality of breast meat originating from slow-growing birds could be achieved by genetic selection on ultimate pH.

As already observed in previous studies [18,19], the initial rate and the extent of decrease in pH are genetically independent. While ultimate pH was shown to be determined by the glycogen content of breast muscle [19,22], strong phenotypic correlations were found between breast meat pH at 15 min *post-mortem* and duration of wing flapping on the shackle line [8]. Moreover, struggling activity after hanging was shown to be more intense and rapid in the slow-growing Label-Rouge line than in heavy or fast-growing lines [9]. We showed for the first time in poultry that this struggling activity is partly genetically determined. According to several studies performed in the context of animal welfare, hanging broilers in an inverted position is experienced as a stressful event [23] associated with increased plasma corticosterone concentrations [9,24]. Vigorous wing flapping is seen as an escape behaviour and an indicator of discomfort. It may be responsible for physical damage such as broken bones or dislocation [25] which may be painful for the animals and responsible for economic losses due to downgrading of carcasses or problems with portioning. As already shown at the phenotypic level [8], and confirmed by the present study at the genetic level, wing flapping also affected breast meat quality by hastening the fall in muscle pH and increasing the redness of the meat, probably because of higher blood flow in the muscles of flapping birds. In addition to improvements in slaughter equipment, this study suggests that the prevalence of wing flapping on the shackle line could be decreased by genetic selection, with positive effects on carcass and meat quality. However, as wing flapping does not entirely summarize bird welfare, the impact on other indicators of stress at slaughter and on the behaviour of birds during rearing should be investigated before any selection can be envisaged.

This study revealed that the genetic correlations between growth, body composition and meat quality traits can differ partially between males and females. As classically observed in the chicken [16], males exhibited a higher growth rate while the females were characterized by earlier development of breast muscle and abdominal fatness, showing that they appear physiologically more mature than males at a given age. Slight differences were also observed in the technological characteristics of the meat. The genetic correlation between the ultimate pH and the lightness of the meat was lower for females than for males. Higher growth rate, associated with greater abdominal fatness, led to a decrease in red colour of the meat in the males while it was not apparent in the females. Although more research is needed to elucidate the mechanisms and the genes relating growth and meat quality in both sexes, our findings suggest some practical recommendations. As males have higher body and breast muscle weight, they appear more appropriate for the cuts market, particularly since meat tenderness increases with muscle growth [19]. Females, which are smaller and have a slightly more acid, pale and exudative meat, would be more appropriate for the whole carcass market.

## Conclusion

The results of this study suggest several relevant criteria for the selection of slow-growing birds in order to adapt their meat to the new market demands. However, such selection is currently made difficult by the fact that the birds have to be killed for the measurement of meat quality, and only sib selection, which is more costly and less efficient, can be applied. Research therefore has to be continued for molecular markers that can be used for the selection of carcass and meat quality- related traits in chickens.

## Competing interests

The authors declare that they have no competing interests.

## Authors' contributions

MC, EB, VG, PC, CA, CB, ELBD contributed to the experimental design. PC and EB performed the lipid measurements. MC, HC, MB and ELBD performed the genetic analyses. All authors read and approved the final manuscript.

## Supplementary Material

Additional file 1 **Table S1.** Heritability estimates (on the diagonal) and genetic correlations (above the diagonal) for body weight, body composition and meat quality traits in males. **Table S2** Heritability estimates (on the diagonal) and genetic correlations (above the diagonal) for body weight, body composition and meat quality traits in females. (DOC 104 kb) Click here for file
