# Supplementary material for: Analysis of a slow-growing line reveals wide genetic variability of carcass and meat quality-related traits
Source: BMC Genet. 2012 Oct 23;13:90. doi: 10.1186/1471-2156-13-90 (PMC3502163; doi:10.1186/1471-2156-13-90)
Supplement: Additional file 1 — Table S1. Heritability estimates (on the diagonal) and genetic correlations (above the diagonal) for body weight, body composition and meat quality traits in males. Table S2 Heritability estimates (on the diagonal) and genetic correlations (above the diagonal) for body weight, body composition and meat quality traits in females. (DOC 104 kb) [file 1471-2156-13-90-S1.doc]

|  | BW3 | BW6 | BW9 | BW12 | BMY | AFP | LEGP | pH15 | pHu | L* | a* | b* | DL | SF | IMF |
| --- | --- | --- | --- | --- | --- | --- | --- | --- | --- | --- | --- | --- | --- | --- | --- |
| BW3 | 0.35 ± 0.12 | **0.87 ± 0.07*** | **0.67 ± 0.14** | 0.37 ± 0.22 | 0.15 ± 0.27 | **0.58 ± 0.17** | -0.08 ± 0.23 | **0.50 ± 0.24** | 0.08 ± 0.22 | 0.06 ± 0.23 | **-0.73 ± 0.17** | -0.16 ± 0.27 | -0.11 ± 0.25 | -0.39± 0.38 | 0.13 ± 0.31 |
| BW6 |  | 0.36 ± 0.11 | **0.9 ± 0.05** | **0.65 ± 0.16** | 0.28 ± 0.28 | **0.60 ± 0.19** | -0.17 ± 0.25 | 0.36 ± 0.27 | 0.16 ± 0.24 | 0.02 ± 0.25 | **-0.74 ± 0.22** | -0.38 ± 0.29 | -0.04 ± 0.27 | -0.50 ± 0.42 | 0.24 ± 0.33 |
| BW9 |  |  | 0.29 ± 0.12 | **0.89 ± 0.06** | 0.32 ± 0.26 | **0.53 ± 0.18** | -0.06 ± 0.23 | 0.21 ± 0.26 | 0.01 ± 0.22 | 0.16 ± 0.22 | **-0.79 ± 0.18** | -0.20 ± 0.28 | 0.05 ± 0.24 | -0.40 ± 0.40 | 0.44 ± 0.28 |
| BW12 |  |  |  | 0.39 ± 0.12 | 0.23 ± 0.26 | **0.43 ± 0.19** | 0.06 ± 0.23 | 0.00 ± 0.26 | -0.13 ± 0.21 | 0.20 ± 0.22 | **-0.71 ± 0.18** | -0.04 ± 0.29 | 0.12 ± 0.24 | -0.22 ± 0.40 | 0.38 ± 0.29 |
| BMY |  |  |  |  | 0.33 ± 0.14 | -0.38 ± 0.22 | -0.05 ± 0 25 | **-0.52 ± 0.27** | -0.02 ± 0.23 | 0.00 ± 0.25 | 0.20 ± 0.28 | 0.05 ± 0.27 | 0.23 ± 0.26 | 0.03 ± 0.42 | -0.08 ± 0.36 |
| AFP |  |  |  |  |  | 0.61 ± 0.21 | -0.27 ± 0.20 | **0.43 ± 0.22** | 0.32 ± 0.19 | -0.09 ± 0.21 | **-0.77 ± 0.15** | -0.38 ± 0.23 | -0.20 ± 0.23 | -0.64 ± 0.38 | 0.27 ± 0.29 |
| LEGP |  |  |  |  |  |  | 0.45 ± 0.12 | 0.03 ± 0.24 | **-0.42 ± 0.19** | 0.31 ± 0.20 | -0.05 ± 0.23 | 0.03 ± 0.24 | -0.10 ± 0.22 | **0.72 ± 0.32** | 0.32 ± 0.27 |
| pH15 |  |  |  |  |  |  |  | 0.31 ± 0.11 | 0.00 ± 0.23 | 0.15 ± 0.23 | **-0.51 ± 0.20** | -0.11 ± 0.28 | -0.14 ± 0.25 | -0.37 ± 0.35 | 0.12 ± 0.32 |
| pHu |  |  |  |  |  |  |  |  | 0.57 ± 0.13 | **-0.90 ± 0.07** | 0.08 ± 0.21 | **-0.59 ± 0.16** | **-0.76 ± 0.12** | -0.33 ± 0.34 | -0.14 ± 0.27 |
| L* |  |  |  |  |  |  |  |  |  | 0.55 ± 0.16 | -0.28 ± 0.21 | **0.46 ±0.20** | **0.84 ± 0.10** | 0.10 ± 0.36 | 0.43 ± 0.28 |
| a* |  |  |  |  |  |  |  |  |  |  | 0.38 ± 0.13 | 0.05 ± 0.27 | -0.01 ± 0.24 | 0.37 ± 0.37 | -0.43 ± 0.31 |
| b* |  |  |  |  |  |  |  |  |  |  |  | 0.34 ± 0.18 | 0.37 ± 0.23 | 0.26 ± 0.41 | -0.27 ± 0.34 |
| DL |  |  |  |  |  |  |  |  |  |  |  |  | 0.43 ± 0.15 | 0.12 ± 0.38 | 0.19 ± 0.31 |
| SF |  |  |  |  |  |  |  |  |  |  |  |  |  | 0.10 ± 0.07 | 0.01 ± 0.48 |
| IMF |  |  |  |  |  |  |  |  |  |  |  |  |  |  | 0.25 ± 0.15 |

**Table S1 Heritability estimates (on the diagonal) and genetic correlations** **(above the diagonal) for body weight, body composition and meat quality traits in males**

| BW3 = Body weight at 3 weeks; BW6 = Body weight at 6 weeks; BW9 = Body weight at 9 weeks; BW12 = Body weight at 12 weeks;  BMY = Breast meat yield; AFP = Abdominal fat percentage; LEGP = Leg percentage; pH15 = pH at 15 min post-mortem; pHu = ultimate pH; | | |
| --- | --- | --- |
| L* = lightness; a* = redness; b* = yellowness; DL = drip loss; SF = shear force; IMF = Intramuscular fat content. * Significant genetic correlations are in bold. |  |  |

**Table S2 Heritability estimates (on the diagonal) and genetic correlations** **(above the diagonal) for body weight, body composition and meat quality traits in females**

|  | BW3 | BW6 | BW9 | BW12 | BMY | AFP | LEGP | pH15 | pHu | L* | a* | b* | DL | SF | IMF |
| --- | --- | --- | --- | --- | --- | --- | --- | --- | --- | --- | --- | --- | --- | --- | --- |
| BW3 | 0.30 ±0.13 | **0.80 ± 0.10*** | **0.72 ± 0.12** | **0.56 ± 0.16** | 0.07 ± 0.24 | 0.16 ± 0.25 | 0.19 ± 0.24 | -0.07 ± 0.27 | -0.17 ± 0.23 | 0.24 ± 0.22 | -0.19 ± 0.25 | -0.10 ±0.27 | -0.17 ± 0.27 | 0.36 ± 0.29 | 0.39 ± 0.34 |
| BW6 |  | 0.32 ±0.14 | **0.93 ±0.04** | **0.82 ± 0.09** | 0.12 ± 0.25 | 0.26 ± 0.24 | 0.15 ± 0.25 | -0.43 ± 0.24 | -0.03 ± 0.24 | 0.09 ± 0.24 | 0.00 ± 0.26 | -0.20 ± 0.28 | -0.20 ± 0.28 | 0.38 ± 0.30 | 0.53 ± 0.32 |
| BW9 |  |  | 0.46 ±0.13 | **0.95 ± 0.03** | 0.14 ± 0.22 | 0.28 ± 0.21 | 0.26 ± 0.22 | -0.30 ± 0.24 | -0.11 ± 0.22 | 0.23 ± 0.21 | -0.28 ± 0.22 | -0.20 ± 0.25 | -0.13 ± 0.26 | 0.43 ± 0.27 | 0.50 ± 0.31 |
| BW12 |  |  |  | 0.60 ± 0.12 | 0.03 ± 0.20 | 0.35± 0.18 | 0.21 ± 0.21 | -0.24 ± 0.22 | -0.23 ± 0.20 | 0.35 ± 0.18 | -0.37 ± 0.20 | -0.19 ± 0.23 | -0.07 ± 0.24 | 0.42 ± 0.25 | 0.49 ± 0.28 |
| BMY |  |  |  |  | 0.52 ± 0.16 | **-0.57 ± 0.15** | 0.23 ± 0.21 | **-0.43 ± 0.22** | 0.25 ± 0.21 | -0.36 ± 0.20 | 0.03 ± 0.22 | -0.10 ± 0.24 | 0.07 ± 0.25 | -0.13 ± 0.29 | -0.51 ± 0.30 |
| AFP |  |  |  |  |  | 0.48 ±0.17 | -0.38 ± 0.20 | 0.26 ± 0.25 | -0.01 ± 0.22 | 0.00 ± 0.21 | -0.25 ± 0.22 | -0.29 ± 0.24 | -0.11 ± 0.26 | -0.31 ± 0.30 | 0.35 ± 0.33 |
| LEGP |  |  |  |  |  |  | 0.48 ±0.15 | 0.10 ± 0.26 | 0.00 ± 0.22 | -0.03 ± 0.21 | -0.34 ± 0.22 | -0.34 ± 0.24 | -0.09 ± 0.26 | **0.65 ± 0.22** | 0.16± 0.36 |
| pH15 |  |  |  |  |  |  |  | 0.30 ± 0.11 | -0.13 ± 0.23 | 0.15 ± 0.23 | **-0.69 ± 0.15** | 0.14 ± 0.27 | -0.31 ± 0.26 | -0.16 ± 0.31 | -0.28 ± 0.37 |
| pHu |  |  |  |  |  |  |  |  | 0.50 ± 0.12 | **-0.78 ± 0.11** | 0.11 ± 0.22 | **-0.57 ± 0.18** | **-0.65 ± 0.18** | **-0.53 ± 0.26** | -0.06 ± 0.33 |
| L* |  |  |  |  |  |  |  |  |  | 0.45 ± 0.13 | -0.27 ± 0.20 | **0.58 ± 0.17** | 0.36 ± 0.22 | **0.57 ± 0.26** | 0.36 ± 0.31 |
| a* |  |  |  |  |  |  |  |  |  |  | 0.42 ± 0.13 | 0.16 ± 0.25 | 0.27 ± 0.25 | -0.03 ± 0.32 | 0.09 ± 0.37 |
| b* |  |  |  |  |  |  |  |  |  |  |  | 0.31 ± 0.15 | 0.27 ± 0.27 | 0.17 ± 0.31 | -0.25 ± 0.39 |
| DL |  |  |  |  |  |  |  |  |  |  |  |  | 0.31 ± 0.12 | 0.24 ± 0.31 | 0.02 ± 0.40 |
| SF |  |  |  |  |  |  |  |  |  |  |  |  |  | 0.17 ± 0.09 | 0.54 ± 0.42 |
| IMF |  |  |  |  |  |  |  |  |  |  |  |  |  |  | 0.16 ± 0.10 |

| BW3 = Body weight at 3 weeks; BW6 = Body weight at 6 weeks; BW9 = Body weight at 9 weeks; BW12 = Body weight at 12 weeks;  BMY = Breast meat yield; AFP = Abdominal fat percentage; LEGP = Leg percentage; pH15 = pH at 15 min post-mortem; pHu = ultimate pH; | | |
| --- | --- | --- |
| L* = lightness; a* = redness; b* = yellowness; DL = drip loss; SF = shear force; IMF = Intramuscular fat content.* Significant genetic correlations are in bold. |  |  |
